# The utility of a network–based clustering method for dimension reduction of imaging and non-imaging biomarkers predictive of Alzheimer’s disease

**DOI:** 10.1038/s41598-018-21118-1

**Published:** 2018-02-12

**Authors:** Hisako Yoshida, Atsushi Kawaguchi, Fumio Yamashita, Kazuhiko Tsuruya

**Affiliations:** 1 Clinical Research Center, Saga University Hospital, Saga, Japan; 2 Section of Clinical Cooperation System, Center for Comprehensive Community Medicine, Faculty of Medicine, Saga University, Saga, Japan; 3 Division of Ultrahigh Field MRI, Iwate Medical University, Yahaba, Japan; 4 Department of Integrated Therapy for Chronic Kidney Disease, Graduate School of Medical Sciences, Kyushu University, Fukuoka, Japan

## Abstract

While the identification of biomarkers for Alzheimer’s disease (AD) is critical, emphasis must also be placed on defining the relationship between these and other indicators. To this end, we propose a network-based radial basis function-sparse partial least squares (RBF-sPLS) approach to analyze structural magnetic resonance imaging (sMRI) data of the brain. This intermediate phenotype for AD represents a more objective approach for exploring biomarkers in the blood and cerebrospinal fluid. The proposed method has two unique features for effective biomarker selection. The first is that applying RBF to sMRI data can reduce the dimensions without excluding information. The second is that the network analysis considers the relationship among the biomarkers, while applied to non-imaging data. As a result, the output can be interpreted as clusters of related biomarkers. In addition, it is possible to estimate the parameters between the sMRI data and biomarkers while simultaneously selecting the related brain regions and biomarkers. When applied to real data, this technique identified not only the hippocampus and traditional biomarkers, such as amyloid beta, as predictive of AD, but also numerous other regions and biomarkers.

## Introduction

Alzheimer’s disease (AD) is the most common form of dementia and is a global problem, especially in developed countries, where the aging population is growing rapidly. Several biomarkers that signal the risk for AD development, such as apolipoprotein E (apoE) ε4 allele^1,2^, before the appearance of dementia symptoms, could play a crucial role in implementing early treatment after a pathological diagnosis of AD based on amyloid beta (Aβ) in the brain tissue^3^.

One of the most popular imaging techniques is structural magnetic resonance imaging (sMRI), which is widely used to examine properties of brain regions. The regression method is one potential way to explore biomarkers, such as hippocampal volume, as a univariate outcome^4^; however, sMRI can also be used to evaluate morphological changes in gray matter density, directly at the voxel level, to classify features related to AD^5–11^. While cognitive and clinical symptoms are the traditional phenotypes (outcomes) for AD, sMRI data are useful as a quantitative intermediate phenotype, which can be evaluated more objectively than traditional phenotypes^12,13^.

MRI data consist of image intensities of millions of voxels in a three-dimensional (3D) array. Since one voxel corresponds to one variable in statistical terms, MRI data are high dimensional. Existing neuroimaging analysis methods require regions of interest (ROI) to be pre-specified in the brain, mainly because of the computational difficulties involved in using a vast number of voxels, while a voxel-based analysis can be used for a more precise analysis. A limitation of this voxel-wise neuroimaging system includes sample sizes that are generally small in comparison with the high dimensionality of the data, making it challenging to define correlations between the response (***Y***) and predictive (***X***) variables. In order to determine the associations among these complex multivariate data sets, partial least squares (PLS), canonical correlation, reduced rank regression, and independent component analyses have been used to detect morphological abnormalities from sMRI data, in association with non-imaging markers^8,10,14,15^.

To analyze sMRI data as an intermediate phenotype and, in parallel, to explore non-imaging biomarkers from over 100 candidates, we have defined multivariate variables for both ***X ***and ***Y*** in a PLS regression model. In our previous study, we proposed the RBF-sPLS approach, which involves application of the radial basis function (RBF) method to 3D sMRI data as a pre-processing step for the sparse partial least squares (sPLS) approach for investigating the relationship between clinical characteristics and brain morphology^16^. Wolz *et al*.^17^ have proposed the nonlinear dimension-reduction method for such studies, although with a limited number of clinical characteristics.

In the present study, we wanted to reduce the dimensionality of non-imaging data as a predictive variable matrix of sPLS in the prediction model of brain morphology. Since the RBF method has the benefit of maintaining the original positional information on brain MRI, even after dimension reduction, it was possible to interpret a region of the brain by re-mapping it onto a brain map from the set of variables extracted after dimension reduction. This was possible as the approach involved a linear (or explicit) formulation as well as a nonlinear relationship, rather than being based solely on a nonlinear function. Furthermore, in this study, we also set out to reduce the dimensions of non-imaging biomarkers as a pre-processing step for the PLS model while simultaneously retaining the information on the relations among variables. However, since the non-imaging biomarkers that we used did not include structural information, such as location information in the brain image, which would have been useful for pre-process dimension reduction, we applied the network-clustering method, which is useful for clarifying structure. Network-based approaches have emerged as powerful tools for studying complex disease models^18^.

To adapt the statistical method for this study, we 1) constructed a network for non-imaging biomarkers (CSF and blood biomarkers) and 2) selected components to simplify the interpretation of diagnostic information. First, the entire network was broken down into sub-networks based on graph theory. For the sPLS algorithm, the summarized values of sub-networks from non-imaging biomarkers were used as ***X***, while radial basis functions were applied to brain imaging data and the resulting values were defined as ***Y***. Although most previous studies using PLS analysis selected the first few components only, we here selected components predictive of disease. For this purpose, we specifically used the score of the PLS as the predictor and the disease status as the response in logistic regression analysis. Thus, the selected components can be interpreted as pairs of brain regions and biomarker networks associated with the AD stage.

The goal of this study was to investigate biomarkers associated with AD using MRI as well as non-imaging data, including the results of blood and CSF sample analyses. To this end, we examined two types of dimension-reduction methods as a pre-procedure for sPLS: the radial basis function method and network-based clustering method for ***X*** and ***Y***, respectively. From the components extracted by the sPLS procedure, we selected certain components to define brain regions and non-imaging biomarkers associated with the incidence of AD, using logistic regression models.We show that the proposed method is particularly useful for achieving dimension reduction, based on typical real-life data.

## Methods and Materials

### Procedure of Network based RBF-sPLS

The network based RBF-sPLS consisted of three steps, as follows:

step 1: Dimension reduction procedure of X and Y, respectively

step 2: Sparse PLS

step 3: Selection of components relevant to AD.

All statistical analyses were performed in R version 3.2.5 by using the following analysis packages: for description of the network graph, [igraph (0.6.5–2)] and [gRapHD (0.2.3)]; for construction of network clustering, [linkcomm (1.0–11)]; for calculation of centralization of clusters, [sna (2.3–2)]; and for multiple imputations for missing data [random Forest (4.6–7)].

#### RBF: Pre-processing for MRI Data Analysis

We used the Statistical Parametric Mapping 8 software (SPM8, Wellcome Department of Cognitive Neurology, London, UK) and the VBM8 toolbox (http://dbm.neuro.uni-jena.de/vbm/download/), running under the MATLAB environment (MathWorks, Natick, MA), to pre-process MR brain images. Three-dimensional T1-weighted images of a subject were first segmented into gray matter, white matter, and CSF space, followed by anatomical normalization to a template image by using DARTEL^19^. The normalized image was finally smoothed with an 8 mm FWHM isotropic Gaussian filter.

#### Dimension Reduction based on Network Clustering for Non-Imaging Data Analysis

As one important facet of this study, we incorporated structural information of non-imaging data, based on network modeling, which might be helpful for interpreting results. This can also reduce the dimensionality of non-imaging data. We performed three steps to achieve this. First, with each non-imaging variable as a vertice (node) of the graph, the network whose edges represented statistical dependence through the decomposition of the joint probability density was estimated using graphical modeling. Second, the vertices of the estimated network were clustered based on similarity in the framework of graphical theory. This is referred to as network clustering, based on a recently developed overlapping clustering approach; Ahn *et al*.^20^ performed pioneering work on overlapping clustering, and Becker *et al*.^21^ developed a new algorithm with their proposed modularity. We used the R package [linkcomm] developed by Kalinka and Tomancak^22^ to implement the method proposed by Becker *et al*.^21^. Third, in order to reduce the dimensionality of the non-imaging data after the clustering procedure, we selected one representative node among each sub-cluster. If more than three nodes belonged to a cluster, we calculated centralization to select the highest information centrality node in each network. This was implemented in the R package [sna]. If only two nodes belonged to one cluster, we calculated the variance of each variable and selected the one with the larger variance as the representative variable of the network. The number of sub-clusters were noted *p*. Thus, we obtained the dimension-reduced *n* × *p* predictive matrix ***X*** for sPLS.

#### Sparse PLS

The PLS regression technique, which was introduced by Wold in 1986^23^, searches for a set of components by using latent variables, and performs a simulation decomposition of ***X*** and ***Y***, with the constraint that these components should explain as much of the covariance between ***X*** and ***Y*** as possible. This was followed by a linear regression step, in which the decomposition of ***X*** is used to predict ***Y***.

In a previous study, we reported on the advantages of RBF by using a simulation method and its application in actual clinical datasets^16^. In this study, we also applied RBF for preprocessing brain data (***Y***_0_). We used the radial B-spline function $\varphi (\cdot )$^24^ to reduce the dimensions, which is represented as follows. For a given *g* ≥ 0,

1 $$\varphi (d)=\frac{1}{4{g}^{2}}\times \{\,\begin{array}{cc}{g}^{3}+3{g}^{2}(g-d)+3g{(g-d)}^{2}-3{(g-d)}^{3}, & (d\le g)\\ {(2g-d)}^{3}, & (g < d\le 2g)\\ 0, & (d > 2g)\end{array}$$

where *d* ≥ 0. We used the distance between these knots to define *g* as $g=\sqrt{3\times {g}_{0}^{2}}$, where ${g}_{0}$ is the distance between adjacent knots. Therefore, ${\boldsymbol{B}}=\varphi (d)$, ${{\boldsymbol{Y}}}_{0}={({{\boldsymbol{Y}}}_{01},\ldots ,{{\boldsymbol{Y}}}_{0n})}^{T}$ and the dependent variable matrix, ***Y***, is constructed as

2 $${\boldsymbol{Y}}={{\boldsymbol{Y}}}_{0}{\bf{B}}$$

The *n* × *q* dimension reduction matrix ***Y*** was defined with the brain image data matrix as the response variable for the sPLS regression analysis.

We defined the response ***Y*** and the predictor ***X*** as follows: the MRI data-reduced dimension by RBF was ***Y***, and the representative variables selected as the most central feature of each cluster of the non-imaging data, such as CSF and demographic data, were ***X***. In PLS regression, the criterion used involved determining the ***Y***-score vector, ${\boldsymbol{u}}={\boldsymbol{Yw}}$, and the ***X***-score vector, ${\boldsymbol{t}}={\boldsymbol{Xv}}$. The weights ***w*** and ***v*** were estimated L(***v***, ***w***) in two subjects to $\Vert {\boldsymbol{v}}{\Vert }_{2}=\Vert {\boldsymbol{w}}{\Vert }_{2}=1$.

3 $${\rm{L}}({\boldsymbol{v}},{\boldsymbol{w}})=-{{\boldsymbol{v}}}^{T}{{\boldsymbol{X}}}^{T}{\boldsymbol{Yw}}+{\lambda }_{{\boldsymbol{X}}}\Vert {\boldsymbol{v}}{\Vert }_{1}+{\lambda }_{{\boldsymbol{Y}}}\Vert {\boldsymbol{w}}{\Vert }_{1}$$

where ${\lambda }_{{\boldsymbol{X}}}$ and ${\lambda }_{{\boldsymbol{Y}}}$ are *L*1 penalization parameters for the weight vectors of matrices ***X*** and ***Y***, respectively. The amplitudes of ${\lambda }_{{\boldsymbol{X}}}$ and ${\lambda }_{{\boldsymbol{Y}}}$ correspond to increases and decreases in the number of ***X*** and ***Y*** variables, which contribute to the regression. The algorithm for the computation of weight vectors ***v*** and ***w*** is as follows:

1. Initialize ***v*** and ***w*** using, for instance, the first pair of singular vectors of the matrix ${{\boldsymbol{X}}}^{T}{\boldsymbol{Y}}$ and normalize ${\boldsymbol{v}}\leftarrow {\boldsymbol{v}}/\Vert {\boldsymbol{v}}{\Vert }_{2}$ and ${\boldsymbol{w}}\leftarrow {\boldsymbol{w}}/\Vert {\boldsymbol{w}}{\Vert }_{2}$.
2. Until convergence of ***v*** and ***w*** with ${h}_{\lambda }(y)=sign(y){(|y|-\lambda )}_{+}$, where ${(a)}_{+}=max(0,a)$.
  - for fixed ${\boldsymbol{w}},\hat{{\boldsymbol{v}}}={h}_{{\lambda }_{{\boldsymbol{X}}}}({{\boldsymbol{X}}}^{T}{\boldsymbol{Y}}{\boldsymbol{w}})$ and then normalize $\hat{{\boldsymbol{v}}}$ as in step 1;
  - for fixed ***v***, $\hat{{\boldsymbol{w}}}={h}_{{\lambda }_{{\boldsymbol{Y}}}}({{\boldsymbol{Y}}}^{T}{\boldsymbol{X}}{\boldsymbol{v}})$ and then normalize $\hat{{\boldsymbol{w}}}$ as in step 1;
  - $${\boldsymbol{v}}=\hat{{\boldsymbol{v}}}$$, $${\boldsymbol{w}}=\hat{{\boldsymbol{w}}}$$.
3. $${\boldsymbol{t}}={\boldsymbol{Xv}}$$, $${\boldsymbol{\ell }}={{\boldsymbol{X}}}^{T}t/{t}^{T}t$$, and $${\boldsymbol{m}}={{\boldsymbol{Y}}}^{T}t/{t}^{T}t$$.
4. The deflation step ${\boldsymbol{X}}\leftarrow {\boldsymbol{X}}-t{{\boldsymbol{\ell }}}^{T}$ and ${\boldsymbol{Y}}\leftarrow {\boldsymbol{Y}}-t{{\boldsymbol{m}}}^{T}$.

By repeating 1) to 4), *k*-th component scores and weights are obtained at the *k*-th repetition. The choice of penalization parameters ${\lambda }_{{\boldsymbol{X}}}$, ${\lambda }_{{\boldsymbol{Y}}}$ and number of components *k* are important in model construction. We fixed the number of components *k *= 10 and used a cross-validation technique with a prediction error sum of squares; PRESS_*jk*_ using the following equation:

4 $$PRES{S}_{j}({\lambda }_{{\boldsymbol{X}}},{\lambda }_{{\boldsymbol{Y}}},k)=\sum _{i=1}^{n}{({y}_{ij}-{\hat{y}}_{(-\kappa (i))j}({\lambda }_{{\boldsymbol{X}}},{\lambda }_{{\boldsymbol{Y}}},k))}^{2}$$

This was applied for the *j*th-dependent variable and the RBF-sPLS model with *k* components defined as follows. Let *κ*: {1, 2, …, n}→{1, 2, …, 5} be an indexing function that indicates the partition to which observation *i* is allocated to $\kappa (i)\,$th part of the data by the randomization. ${\hat{y}}_{(-\kappa (i))j}({\lambda }_{{\boldsymbol{X}}},{\lambda }_{{\boldsymbol{Y}}},k)$ is the predicted value for the *j*th dependent variable from the sPLS model with penalization parameters ${\lambda }_{{\boldsymbol{X}}}$ and ${\lambda }_{{\boldsymbol{Y}}}$ and the number of components *k* and estimated weight vectors from $\kappa (i)\,$th part of the data removed. That is, for any *i* subject, we predict that ${\hat{y}}_{(-\kappa (i))j}({\lambda }_{{\boldsymbol{X}}},{\lambda }_{{\boldsymbol{Y}}},k)={x}_{i}{\hat{b}}_{(-\kappa (i))j}({\lambda }_{{\boldsymbol{X}}},{\lambda }_{{\boldsymbol{Y}}},k)$, where ${\hat{b}}_{(-\kappa (i))j}({\lambda }_{{\boldsymbol{X}}},{\lambda }_{{\boldsymbol{Y}}},k)$ is the *j*th column of estimated regression coefficient matrix *C* from the RBF-sPLS model with penalization parameters ${\lambda }_{{\boldsymbol{X}}}$ and ${\lambda }_{{\boldsymbol{Y}}}$ and number of components *k* and $\kappa (i)\,$th part of the data removed. We selected the optimal set $({\lambda }_{{\boldsymbol{X}}},{\lambda }_{{\boldsymbol{Y}}})$ based on the binary search with $PRES{S}_{j}({\lambda }_{{\boldsymbol{X}}},{\lambda }_{{\boldsymbol{Y}}},k)$ as the objective function.

#### Component Selection

In normal PLS analyses, the number of components *k* is selected with the first *k* components using criteria such as the cross-validation error. These criteria, for example Q^2^ as proposed by Lê Cao^25^, can estimate *k* (the number of PLS components). In the present study, we opted for setting 10 components as *k* with reference to our experience and based on previous reports. If this analysis was applied, subcomponents of ***X*** and ***Y*** would be related within each component, but they would not be related to the outcome (i.e., they would be independent of whether AD is present). To address this, we applied a backward stepwise-selection based on the multivariable logistic regression model with the $n\times k$ score matrix ***T*** of the PLS algorithm as predictors and the AD diagnosis at baseline as the response variable. The Bayesian information criterion (BIC) was used to select the optimal model corresponding to the selected components. Thus, the obtained components are the set of brain regions and networks of non-imaging biomarkers relevant to a diagnosis of AD.

### Application to ADNI Data

#### Blood/CSF Biomarkers and Brain Imaging Data of ADNI

Data used in the preparation of this article were obtained from the Alzheimer’s Disease Neuroimaging Initiative (ADNI) database (adni.loni.usc.edu). As such, the investigators within the ADNI contributed to the design and implementation of ADNI and/or provided data, but did not participate in analysis or writing of this report. The ADNI was launched in 2003 by a 5-year public-private partnership of several research institutions and private pharmaceutical companies.

This ADNI project was approved by the Institutional Review Boards of all the participating institutions. Informed written consent was obtained from all participants at each site. The ADNI database includes results from various types of imaging examinations and from clinical and neuropsychological assessments. Accordingly, meaningful results can likely be obtained by compiling this information to measure the progression of mild cognitive impairment (MCI) and early AD.

To analyze brain imaging data, we used T1-weighted MR images from the ADNI1 database. MRI acquisition had been performed according to the ADNI acquisition protocol^26^. CSF, proteomic, and blood-sample biomarkers obtained via peripheral veins and these, along with vital signs were defined as non-imaging biomarkers. First, we screened 398 non-imaging biomarkers in AD, MCI, and normal control (NC) individuals, as defined by ADNI criteria. In a number of cases, participants refused to give consent for the more invasive examinations, such as lumbar puncture; thus, CSF markers were not available for all, particularly in asymptomatic cases. We applied the “random forest” method^27^, which replaces missing values by using the R package [randomForest] and created a new data set (*X*_0_) by excluding cases with missing items in the examinations. The first step of the rfImpute method creates various subsets randomly using bootstrap methods, while the proximity matrix from these subsets is used to update the imputation of missing values.

#### Numerical Evaluation

We used an already well-studied dataset for the evaluation. In addition, we numerically evaluated the efficacy and stability of the result focusing on the network-based cluster analysis, which was newly applied in our analysis. Since this cluster analysis was used as the dimension-reduction method for the sPLS analysis, in order to focus on the effect of the clustering algorithm, we simplified the sPLS using a univariate response (the ratio of the hippocampal volume to the intra cranial volume). This corresponds to multiple regression analysis with representative variables of each cluster group in ***X*** extracted by each clustering as independent variables. This multiple regression analysis with L1 penalization and backward stepwise variable selection was repeated 1000 times using the resampling technique, and we calculated adjusted r-squared values and mean-squared error (MSE) from bootstrap samples that did not contain that observation for each regression model. The mean r squared values and MSE among repeats were compared among the three clustering algorithms: network clustering (our approach), hierarchical clustering^28^, and mixture distribution model^29^. These methods have been applied for some biomarker studies^30,31^.

## Results

### Pre-processing for MRI Data and Non-Imaging Biomarkers

In the present study, 534 baseline MRI scans at 1.5T were downloaded from the ADNI database for evaluation in March 2013; these consisted of 104 AD and 430 non-AD (NC + MCI) scans. The demographical baseline data of all subjects are presented in Table 1. First, we examined the dimension reduction procedure with respect to the response variable. Out of the 2,122,945 (121 × 145 × 121) voxels for each subject, all voxels representing grey matter were extracted, resulting in 839,089 voxels. The dimension of the basis function was *q* = 7,176, because the knots were equally distributed with a 4-voxel interval (*h*_0_ = 4; therefore, $h=\sqrt{3\,\times \,{4}^{2}}=6.93$). Thus, the $n\times q$ brain data matrix ***Y*** was created.

**Table 1:** Demographical characteristics and laboratory data.

	NC (*n* = 57)	MCI (*n* = 373)	AD (*n* = 104)
Age (years)	75 ± 6	75 ± 7	75 ± 8
Male sex, *n* (%)	29 (51)	243 (64)	60 (61)
Smoking habits, *n* (%)	29 (51)	157 (42)	41 (37)
Alcohol abuse, *n* (%)	4 (7.0)	15 (4.0)	6 (6.0)
Previous history: hypertension, *n* (%)	28 (49)	183 (48)	56 (53)
Previous history: stroke, *n* (%)	1 (1.8)	7 (1.9)	2 (1.9)
SBP (mmHg)	131 ± 18	133 ± 16	135 ± 17
DBP (mmHg)	74 ± 8	74 ± 9	74 ± 9
BMI (kg/m2)	27.0 ± 4.2	26.1 ± 3.9	25.6 ± 3.9
Apo E ε4 allele	0 (0)	45 (12)	22 (21)
CDR	0 (0–0)	1.5 (1–2)	4.5 (3.5–5)
MMSE	29 (28–30)	27 (25–29)	24 (22–25)
Family history of AD	15 (26)	94 (25)	30 (28)
Medication *n* (%)	0 (0.0)	170 (45.0)	99 (93.4)

Data are expressed as mean ± SD or median (interquartile range) for continuous variables, and as number (percentage) for categorical variables.

Abbreviations: SBP, systolic blood pressure; DBP, diastolic blood pressure; BMI, body mass index; Apo E, Apolipoprotein E; CDR, Clinical Dementia Rating; MMSE, mini-mental state examination; AD, Alzheimer’s disease

Medication: participants who took medication for AD at baseline, such as donepezil (Aricept^®^), rivastigmine (Exelon^®^), galantamine (Razadyne^®^, Reminyl^®^), memantine (Namenda^®^).

**Figure 1:**
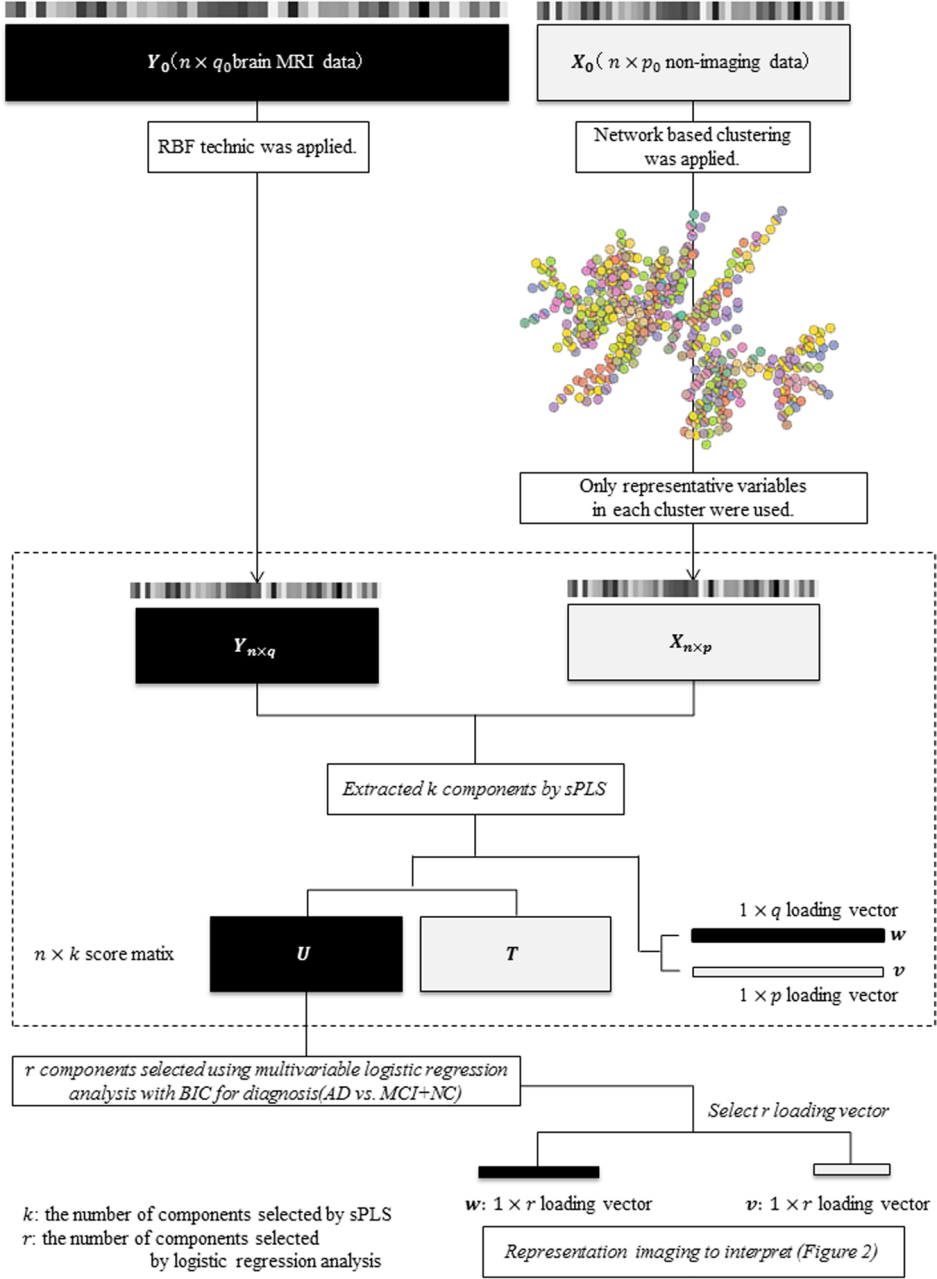
Schematic diagram of the study. We applied a network-based RBF-sPLS to the ADNI database. RBF was applied for dimension reduction of neuroimaging data, and network clustering was similarly applied for non-imaging biomarkers. Notations: *k*, number of components selected by sPLS; *r*, number of components selected by a multiple logistic regression analysis in their final forms.

A total of 398 non-imaging blood and CSF biomarkers, including the apoE ε4 allele, were screened as covariates (*p*_0_ = 398) and 209 clusters were constructed. The network for each cluster was constructed from extensively overlapping nodes. The largest network cluster grew to 12 vertices (variables) and the smallest one grew to two vertices. This network is illustrated in Fig. 1; nodes from different networks are represented by different colors. We created a new data set with 165 (the notation was *p*) representative variables as the center variable of each cluster, after the overlapping variables were combined into one. Thus, the $n\times p$ biomarker matrix ***X*** was defined.

### Sparse Partial Least Squares and Component Selection

Next, we estimated the Q^2^ criterion^25^, which was defined by the residual and prediction error sum of squares in the PLS model and indicated the correlations between sub-components of ***X*** and ***Y***. According to Q^2^ criterion, we found that the number of optimal PLS components was 2. However, these 2 components were not necessarily related to AD and could not to identify components relevant to AD, because the sPLS procedure did not utilize the AD diagnosis information. Thus, to select components relevant to AD from a larger number of components by means of logistic regression, we selected 10 sets of initial components as a complementary analysis to the PLS. We assessed how the results changed according to the number of initial components, namely a sensitivity analysis was conducted (Supplemental Fig. 1). For the sPLS algorithm, the optimal ${\lambda }_{{\boldsymbol{X}}}$ and ${\lambda }_{{\boldsymbol{Y}}}$ were chosen (${\lambda }_{{\boldsymbol{X}}}=0.123$. ${\lambda }_{{\boldsymbol{Y}}}=0.012$). Each component contained 547 to 1154 non-zero voxels and one to seven biomarkers. Of note, brain images were represented as “regions” comprised of neighboring voxels, and components of ***X*** were represented as a set of biomarkers constructed from the network. To select the components relevant to AD, we applied a logistic regression analysis using the score of each ***Y***’s component (the notation was ***u***) as explanation variables, and diagnosis (AD = 1, not AD = 0) as a response variable.

**Figure 2:**
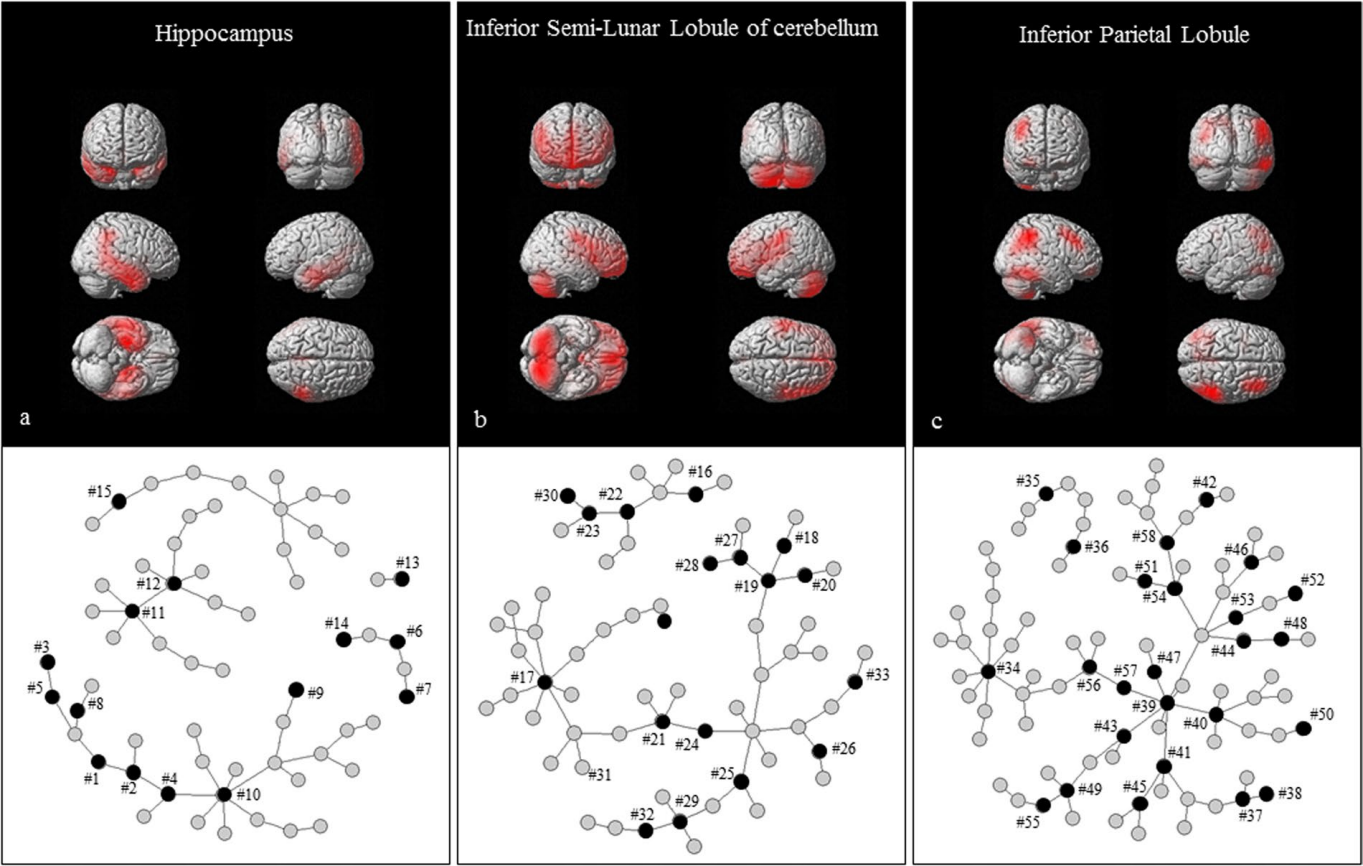
Three components selected by RBF-sPLS. (a) The most significant component (*p* value = 4.19 × 10^−15^) for Alzheimer’s disease, as identified by a logistic regression analysis, were derived from 10 pairs of brain regions and biomarkers extracted using RBF-sPLS. The red color on the brain images indicates significant atrophied areas, especially the hippocampi and left temporal lobe. The black circles on the network graph indicate representative variables of this component, and the gray circles indicate the nodes correlated with these representative variables. The numbers (#) on the network area match the numbers in Table 2. (b,c) The second and third most significant components (*p* value = 6.34 × 10^−4^ for b, *p* value = 1.41 × 10^−4^ for c) for Alzheimer’s disease are displayed analogous to (a). The numbers (#) on the network area match the numbers in Supplemental Table 1a,b.

Three components were selected as relevant sets of brain regions and biomarkers for AD (Fig. 2a–c) by logistic regression, using BIC. The third of the first set of 10 components corresponded to both hippocampi, as well as to the apoE ε4 allele (indicated as #8 and #5 in Table 2 and Fig. 2a) and 14 other protein markers: phosphorylated tau 181P (#1), total tau (#2), apoE protein (#3), heart fatty acid-binding protein (#4), chitinase 3-like 1 (#6 and #7), gamma enolase (#9), pyruvate kinase M1/M2 (#10), peroxiredoxin-2 (#11 and #12), percent of neutrophils (#13), glial fibrillary acidic protein (#14), and body mass index (#15). These biomarkers were the representative variables for each cluster. At least one biomarker was present among these variables in each cluster (Table 2). In addition, two other brain regions and a set of non-imaging biomarkers linked with these brain regions were selected (Fig. 2b,c; Supplemental Table 2a,b, #16 to #58).

**Table 2:** Significant brain region and biomarkers for AD.

	Representative variable of cluster; ***v*** is loading for X./Markers in each cluster
#1	phosphorylated tau 181 P; ***v***-0.507 and had 2 clusters
	Amyloid beta 142, Genotype - Allele 1, Apolipoprotein-E
	Total tau
#2	total tau; ***v*** −0.484 and had 3 clusters
	Fatty acid-binding protein heart, Pyruvate kinase isozymes M1//M2, Fructose-bisphosphate aldolase A
	Tau
	Phosphorylated tau 181p
#3	Apolipoprotein-E protein; ***v*** −0.352 and had 1 cluster
	Genotype - allele 2
#4	Fatty acid-binding protein, heart; ***v*** −0.341 and had 2 clusters
	Fatty acid-binding protein, heart, Pyruvate kinase isozymes M1//M2, Protein FAM3C, Cytoplasmic, Neuroblastoma suppressor of tumorigenicity 1, Aspartate aminotransferase, Cytoplasmic, Aspartate aminotransferase, ProSAAS, Fructose-bisphosphate aldolase A, Beta-2-microglobulin
	Pyruvate kinase isozymes M1//M2, Fructose-bisphosphate aldolase A
#5	Genotype - Allele 2; ***v*** −0.335 and had 2 clusters
	Amyloid beta 142
	Apolipoprotein-E
#6	Chitinase-3-like protein 1;***v*** −0.223 and had 2 clusters
	Vasorin
	Chitinase-3-like protein 1
#7	Chitinase-3-like protein 1; ***v*** −0.207 and had 1 cluster
	Chitinase-3-like protein 1
#8	Genotype - Allele 1; ***v*** −0.169 and had 4 clusters
	Phosphorylated tau 181p, Amyloid beta142, Apolipoprotein-E
#9	Gamma-enolase; ***v*** −0.019 and had 1 cluster
	Aspartate aminotransferase, Mitochondrial
#10	Pyruvate kinase isozymes M1//M2; ***v*** −0.063 and had 4 clusters
	Alpha-1-antitrypsin, Alpha-1-antitrypsin, Aspartate aminotransferase, Cytoplasmic
	Fatty acid-binding protein heart, Fatty acid-binding protein heart, Protein FAM3C, cytoplasmic, Neuroblastoma suppressor of tumorigenicity 1, Aspartate aminotransferase, Cytoplasmic, Aspartate aminotransferase, ProSAAS, Fructose-bisphosphate aldolase A, Beta-2-microglobulin
	Fatty acid-binding protein heart, Fructose-bisphosphate aldolase A
	Aspartate aminotransferase, Cytoplasmic, Aspartate aminotransferase, Mitochondrial, Osteopontin, Apolipoprotein D, Brain acid soluble protein 1
#11	Peroxiredoxin-1; ***v*** −0.060 and had 4 clusters
	Hemoglobin subunit alpha, Hemoglobin subunit alpha, Hemoglobin subunit beta, Peroxiredoxin-2, Peroxi-redoxin-6
	Neural cell adhesion molecule L1, Neural cell adhesion molecule L1, Neural cell adhesion molecule L1
	Peroxiredoxin-1, Peroxiredoxin-2, Peroxiredoxin-6
	Peroxiredoxin-2, Peroxiredoxin-2, Transforming growth factor beta-1, Hemoglobin in CSF, Catalase
#12	Peroxiredoxin-2; ***v*** −0.044 and had 3 clusters
	Hemoglobin subunit alpha, Hemoglobin subunit alpha, Hemoglobin subunit beta, Peroxiredoxin-1, Peroxiredoxin-6
	Peroxiredoxin-1, Peroxiredoxin-1, Peroxiredoxin-6
	Peroxiredoxin-1, Peroxiredoxin-2, Transforming growth factor beta-1, Hemoglobin in CSF, Catalase
#13	Neutrophils (%); ***v*** −0.025 and had 1 cluster
	Lymphocytes (%)
#14	Glial fibrillary acidic protein; ***v*** −0.134 and had 1 cluster
	Vasorin
#15	Body mass index at baseline; ***v*** 0.077 and had 2 clusters
	Sex, Platelets, White blood cell, Triglycerides, Total cholesterol, Creatinine, Uric acid, Phosphorus, Body weight at baseline, Body weight at screening time, Height at screening time
	Body mass index at screening time

Fifteen representative variables of X contained in the most significant component and related variables in each cluster are shown. The numbers (#) in the table match the number values in Fig. 2a. The loading of each variable is indicated in this table as ***v***. Because biomarkers are identified by different antibodies, there can be two or more biomarkers with same name.

### The Efficacy of the Network-based RBF-sPLS Strategy

In order to show the benefits of our method, we compared three variations of clustering and dimension-reduction methods using a resampling technique, as a sensitivity analysis. The result of repeating 1,000 times in each clustering method, the median of the *r*-squared value was larger in our proposed method than in hierarchical clustering and mixture distribution model (Supplemental Fig. 2). These *r*-squared values indicated how well the representative variables, selected from each clustering method, fit the regression model as independent variables. The MSEs from bootstrap samples not containing that observation for each regression model, i.e., the distance between an estimator and the true underlying parameter, were sufficiently small and were less than those obtained by two other methods. Thus, more useful variables for the regression model were selected in our network clustering-based dimension-reduction method.

## Discussion

The present study describes the application of the RBF-sPLS technique to performing a high-dimensional regression analysis, to assess correlations between brain MRI data and non-imaging biomarkers, from the ADNI database. Our proposed method, the network-based RBF-sPLS, applies two dimension-reduction methods: RBF and network clustering, as a pre-procedure for PLS. The advantage of these two dimension-reduction methods is that it allows the results from PLS to be more interpretable.

The strengths of the present study are as follows. First, we have newly applied the network-based RBF-sPLS technique for a high-dimensional regression analysis. We reduced the number of parameters without losing information about the correlation between non-imaging biomarkers using a network-clustering method, while preserving the advantages of RBF-sPLS that have been previously reported. In the present study, our proposed method showed results with high interpretability for AD. Although not assessed in detail, our method may be more stable in terms of a defined representative value for the predicting model than classical clustering methods, such as hierarchical clustering. Second, since we defined the network of biomarkers, the resulting components (i.e., the pairs of brain images and non-imaging biomarkers) can be interpreted not only based on the representative biomarkers used in the PLS model, but also by other biomarkers related to these representative biomarkers. Thus, this method would unearth a great deal of information for medical consideration. In addition, the traditional biomarkers, such as the apoE ε4 allele, tau, and several other proteins, were included as components significantly predicting AD. The network clustering suggests an indirect correlation between AD and these proteomic variables. Accordingly, through this strategy, it may be possible to identify the real markers for a disease that is initially diagnosed by a surrogate marker.

There are several limitations in the present study. First, not all of the participants’ baseline data from ADNI were used. This is primarily because samples from invasive examinations, such as lumbar puncture, were often not obtained. However, ADNI provides various different strategies for estimating missing data. A few methods for replacing missing values as block units have recently been proposed^32,33^. Therefore, in future studies, we will attempt to implement this technique to complete the database. Further, we focused on only the difference between AD and not-AD (MCI + NC), for the purpose of evaluating the approach. We did not attempt to distinguish between other combinations of AD stages (*i.e*. AD *vs*. MCI, MCI *vs*. NC). Our method could be extended to a multinomial response model, such as AD *vs.* MCI *vs*. NC according to a clinical interest, but this is the different scope of this paper. We will address this in future.

Second, this study employed a cross-sectional analysis. We relied on diagnoses that were based on tests of cognitive function at the time of screening, but a definitive diagnosis based on pathology was not available. Thus, we cannot exclude the possibility that the diagnoses used in this study might include misclassifications. The ADNI database also contains the following longitudinal data: ADNI1, ADNI2, and ADNIGO, which are valuable databases, because they contain long-term observations over the course of as much as 10 years. While we did not perform a longitudinal analysis, the RBF-sPLS technique could be applied effectively to such an analysis, thereby including a time-dependent analysis of the progression of AD, from onset or MCI to AD.

Third, we need to consider the method used to define the representative variables for each network and the method used to select the number of initial components for sPLS. After testing several methods for this study, the representative variables were defined using information centrality, and we extracted 10 sPLS components for logistic analysis. Because the purpose of present study was to investigate the effect of dimension reduction using a network model, the methods by which to select the representative variables and the number of sPLS components were out of the scope of the study. However, in future studies we plan to use the relationship described by the degrees between the network and the diagnosis, according to graph theory. Thereafter, we would like to investigate the robustness of our proposed method as only the minimal metric experiment was performed in the present study.

## Conclusions

We here applied a network-based RBF-sPLS technique to identify several brain regions and non-imaging biomarkers that were related to AD, simultaneously.

## Electronic supplementary material

Supplemental Information
