# Supplementary material for: The utility of a network–based clustering method for dimension reduction of imaging and non-imaging biomarkers predictive of Alzheimer’s disease
Source: Sci Rep. 2018 Feb 12;8:2807. doi: 10.1038/s41598-018-21118-1 (PMC5809402; doi:10.1038/s41598-018-21118-1)
Supplement: Supplementary file 1 — Supplemental Information [file 41598_2018_21118_MOESM1_ESM.pdf]

The utility of a network–based clustering method for dimension reduction of imaging and non-imaging biomarkers predictive of Alzheimer's disease

Hisako Yoshida [1)], Atsushi Kawaguchi [2)], Fumio Yamashita [3)], Kazuhiko Tsuruya [4)]

1) Clinical Research Center, Saga University Hospital, Saga, Japan
   E-mail: hyoshida@cc.saga-u.ac.jp
2) Section of Clinical Cooperation System, Center for Comprehensive Community Medicine, Faculty of Medicine, Saga University, Saga, Japan
   E-mail: akawa@cc.saga-u.ac.jp
3) Division of Ultrahigh Field MRI, Iwate Medical University, Yahaba, Japan
   E-mail: fyamashi@iwate-med.ac.jp
4) Department of Integrated Therapy for Chronic Kidney Disease, Graduate School of Medical Sciences, Kyushu University, Fukuoka, Japan
   E-mail: tsuruya@intmed2.med.kyushu-u.ac.jp

Supplemental Table 1a. Significant biomarkers for the diagnosis of AD and other markers in each network.

	Representative variable of cluster; v is loading for X. /Markers in each cluster
#16	age; v 0.639 and had 1 cluster
	Insulin-like growth factor-binding protein 2, Latent-transforming growth factor beta-binding protein 2, Diastolic blood pressure at screening time
#17	sex; v 0.452 and had 2 clusters
	Hemoglobin, Calcium, Total cholesterol, Creatinine, Alanine transaminase, Urea nitrogen, Pulse rate at baseline
	Platelets, Red blood cells, Triglycerides, Total cholesterol, Creatinine, Uric acid, Phosphorus, Body weight at baseline, Body weight at screening time, Height at screening time, Body mass index
#18	Alpha-1-antichymotrypsin; v 0.289 and had 2 clusters
	Complement C3
	Alpha-1-antichymotrypsin
#19	Alpha-1-antichymotrypsin; v 0.264 and had 4 clusters
	Complement component C6, Alpha-1B-glycoprotein, Vitamin D-binding protein, Ceruloplasmin, Complement factor B
	Leucine-rich alpha-2-glycoprotein
	Alpha-1-antichymotrypsin
	Alpha-1-antichymotrypsin
#20	Alpha-1-antichymotrypsin; v 0.264 and had 2 clusters
	Complement C3
	Alpha-1-antichymotrypsin
#21	Protein AMBP; v 0.200 and had 2 clusters
	Complement C3, Protein AMBP, Creatinine
	Protein AMBP, Vitamin D-binding protein, Abeta1-42 result in plasma
#22	Insulin-like growth factor-binding protein 2; v 0.182 and had 1 cluster
	Insulin-like growth factor-binding protein 2, Kallikrein-10, Mimecan, Mimecan, Metalloproteinase inhibit-or 1
#23	Metalloproteinase inhibitor 1; v 0.144 and had 3 clusters
	Insulin-like growth factor-binding protein 2, Insulin-like growth factor-binding protein 2, Kallikrein-10, Mimecan, Mimecan
	Complement C1q subcomponent subunit B
	Metalloproteinase inhibitor 1

#24	Protein AMBP; v 0.140 and had 1 cluster
	Protein AMBP, Vitamin D-binding protein, Abeta1-42 result in plasma
#25	Hemopexin; v 0.131 and had 1 cluster
	Hemopexin, Hemopexin, Vitamin D-binding protein, Vitamin D-binding protein, Protein in the CSF
#26	N-acetylmuramoyl-L-alanine amidase; v 0.099 and had 1 cluster
	Alpha-2-HS-glycoprotein, N-acetylmuramoyl-L-alanine amidase, Prothrombin
#27	Leucine-rich alpha-2-glycoprotein; v 0.084 and had 3 clusters
	C-reactive protein
	Alpha-1-antichymotrypsin
	Leucine-rich alpha-2-glycoprotein
#28	Leucine-rich alpha-2-glycoprotein; v 0.072 and had 1 cluster
	Leucine-rich alpha-2-glycoprotein
#29	Hemopexin; v 0.071 and had 4 clusters
	Complement C2, Complement C2, Complement C2
	Fibromodulin
	Hemopexin, Hemopexin, Vitamin D-binding protein, Vitamin D-binding protein, protein
	Apolipoprotein B-100
#30	Metalloproteinase inhibitor 1; v 0.062 and had 1 cluster
	Metalloproteinase inhibitor 1
#31	Complement factor B; v 0.042 and had 3 clusters
	Complement component C6, Alpha-1B-glycoprotein, Alpha-1-antichymotrypsin, Vitamin D-binding protein, Ceruloplasmin
	Complement factor B
	Complement factor B
#32	Complement C2; v 0.019 and had 1 cluster
	Complement C2, Complement C2, Hemopexin
#33	Alpha-2-HS-glycoprotein; v 0.013 and had 2 cluster
	Alpha-2-HS-glycoprotein
	Alpha-2-HS-glycoprotein

Supplemental Table 1b. Significant biomarkers for the diagnosis of AD, and other markers in each network.

	Representative variable of cluster, v is loading for X. /Markers in each cluster
#34	Sex; v 0.406 and had 2 clusters
	Hemoglobin, Calcium, Total cholesterol, Creatinine, Alanine transaminase, Urea nitrogen, Pulse rate at baseline
	Platelets, White blood cell, Triglycerides, Total cholesterol, Creatinine, Uric acid, Phosphorus, Body weight at baseline, Body weight at screening time, Height at screening time, Body mass index at baseline
#35	Age; v 0.366 and had 1 cluster
	Insulin-like growth factor-binding protein 2, Latent-transforming growth factor beta-binding protein 2, Diastolic blood pressure at screening time
#36	Respiratory rate at baseline; v 0.083 and had 2 clusters
	Systolic blood pressure at baseline, Systolic blood pressure at screening time, Diastolic blood pressure at screening time
	Respiratory rate at screening time
#37	Leucine-rich alpha-2-glycoprotein; v -0.401 and had 3 clusters
	C-reactive protein
	Alpha-1-antichymotrypsin
	Leucine-rich alpha-2-glycoprotein
#38	Leucine-rich alpha-2-glycoprotein; v -0.381 and had 1 cluster
	Leucine-rich alpha-2-glycoprotein
#39	Vitamin D-binding protein; v -0.247 and had 6 clusters
	Complement component C6, Complement component C6, Vitamin D-binding protein, Ceruloplasmin, Ceruloplasmin
	Complement component C6, Alpha-1B-glycoprotein, Alpha-1-antichymotrypsin, Ceruloplasmin, Complement factor B
	Hemopexin, Hemopexin, Vitamin D-binding protein, protein
	Kininogen-1, Plasminogen, Plasminogen
	N-acetylmuramoyl-L-alanine amidase,
	Protein AMBP, Protein AMBP, Abeta1-42 result in plasma
#40	Kininogen-1; v -0.244 and had 3 clusters
	Kininogen-1, Afamin, Afamin
	Plasminogen, Plasminogen, Vitamin D-binding protein
	Kininogen-1, Plasminogen, Plasminogen
#41	Complement component C6; v -0.194 and had 2 clusters
	Complement component C6, Vitamin D-binding protein, Vitamin D-binding protein, Ceruloplasmin, Ceruloplasmin
	Alpha-1B-glycoprotein, Alpha-1-antichymotrypsin, Vitamin D-binding protein, Ceruloplasmin, Complement factor B
#42	Inter-alpha-trypsin inhibitor heavy chain H1; v -0.191 and had 2 clusters
	Complement C5, Complement C5, Complement C5, Exostosin-like 2, Kallistatin, factor H, Monocyte differentiation antigen CD14
	Inter-alpha-trypsin inhibitor heavy chain H1
#43	Hemopexin; v -0.175 and had 1 cluster
	Hemopexin, Hemopexin, Vitamin D-binding protein, Vitamin D-binding protein, Protein in CSF
#44	Alpha-2-HS-glycoprotein; v -0.157 and had 2 clusters
	Alpha-2-HS-glycoprotein
	N-acetylmuramoyl-L-alanine amidase, N-acetylmuramoyl-L-alanine amidase, Prothrombin
#45	Complement factor B; v -0.154 and had 3 clusters
	Complement component C6, Alpha-1B-glycoprotein, Alpha-1-antichymotrypsin, Vitamin D-binding protein , Ceruloplasmin

		Complement factor B
		Complement factor B
#46	Complement component C8 beta chain; v -0.151 and had 2 clusters	
		Complement C3, Complement component C8 beta chain, Complement component C8 beta chain
		Complement component C8 beta chain, N-acetylmuramoyl-L-alanine amidase, Secretogranin-1
#47	Alpha-1B-glycoprotein; v -0.071 and had 2 clusters	
		Alpha-1B-glycoprotein
		Complement component C6, Alpha-1-antichymotrypsin, Vitamin D-binding protein, Ceruloplasmin , Complement factor B
#48	Alpha-2-HS-glycoprotein; v -0.133 and had 2 clusters	
		Alpha-2-HS-glycoprotein
		Alpha-2-HS-glycoprotein
#49	Hemopexin; v -0.132 and had 4 clusters	
		Complement C2, Complement C2, Complement C2
		Fibromodulin
		Hemopexin , Hemopexin, Vitamin D-binding protein, Vitamin D-binding protein , Protein in CSF
		Apolipoprotein B-100
#50	Afamin; v -0.111 and had 1 cluster	
		Afamin
#51	Kallistatin; v -0.111 and had 2 clusters	
		Kallistatin
		Kallistatin
#52	Prothrombin; v -0.085 and had 1 cluster	
		Prothrombin
#53	N-acetylmuramoyl-L-alanine amidase; v -0.085 and had 1 cluster	
		Alpha-2-HS-glycoprotein, N-acetylmuramoyl-L-alanine amidase, Prothrombin
#54	Kallistatin; v -0.046 and had 4 clusters	
		Complement C5, Complement C5, Complement C5, Exostosin-like 2, Inter-alpha-trypsin inhibitor heavy chain H1, Factor H
		Kallistatin
		Kallistatin
		N-acetylmuramoyl-L-alanine amidase
#55	Complement C2; v -0.025 and had 1 cluster	
		Complement C2, Complement C2, Hemopexin
#56	Protein AMBP; v -0.018 and had 2 clusters	
		Complement C3, Protein AMBP, Creatinine
		Protein AMBP, Vitamin D-binding protein, Abeta1-42 result in plasma
#57	Protein AMBP; v -0.015 and had 1 cluster	
		Protein AMBP, Vitamin D-binding protein, Abeta1-42 result in plasma
#58	Complement C5; v -0.006 and had 1 cluster	
		Complement C5, Complement C5, Exostosin-like 2, Inter-alpha-trypsin inhibitor heavy chain H1, Kallistatin, factor H, Monocyte differentiation antigen CD14

The representative variables of X contained in the second (Supplemental Table 1a) and third (Supplemental Table 1b) significant components and related variables in each cluster are shown. The numbers (#) in Supplemental Table 1a and 1b match the number values in Figure 2b and 2c,

respectively. The loading of each variable is indicated in this table as v. Because biomarkers are identified by different antibodies, there were two or more biomarkers with same name

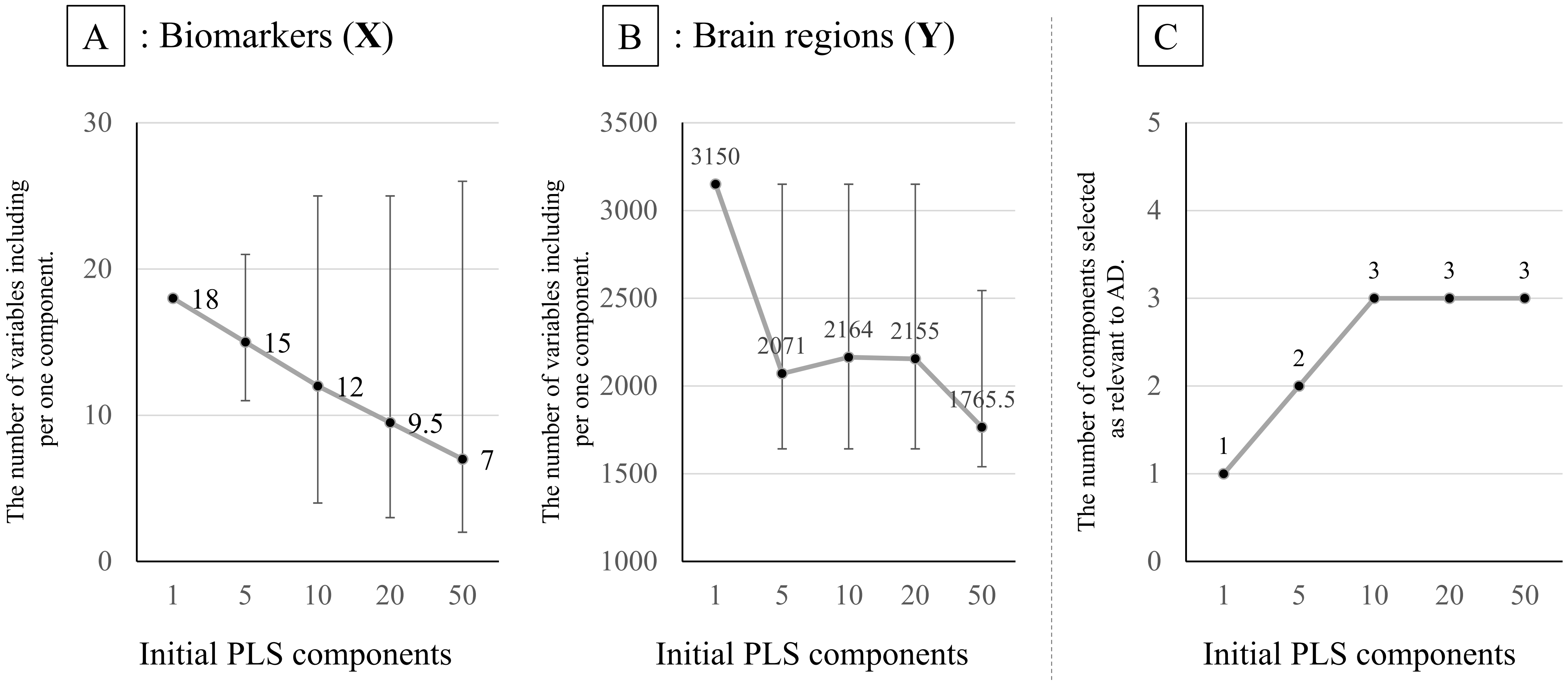

Supplemental Figure 1

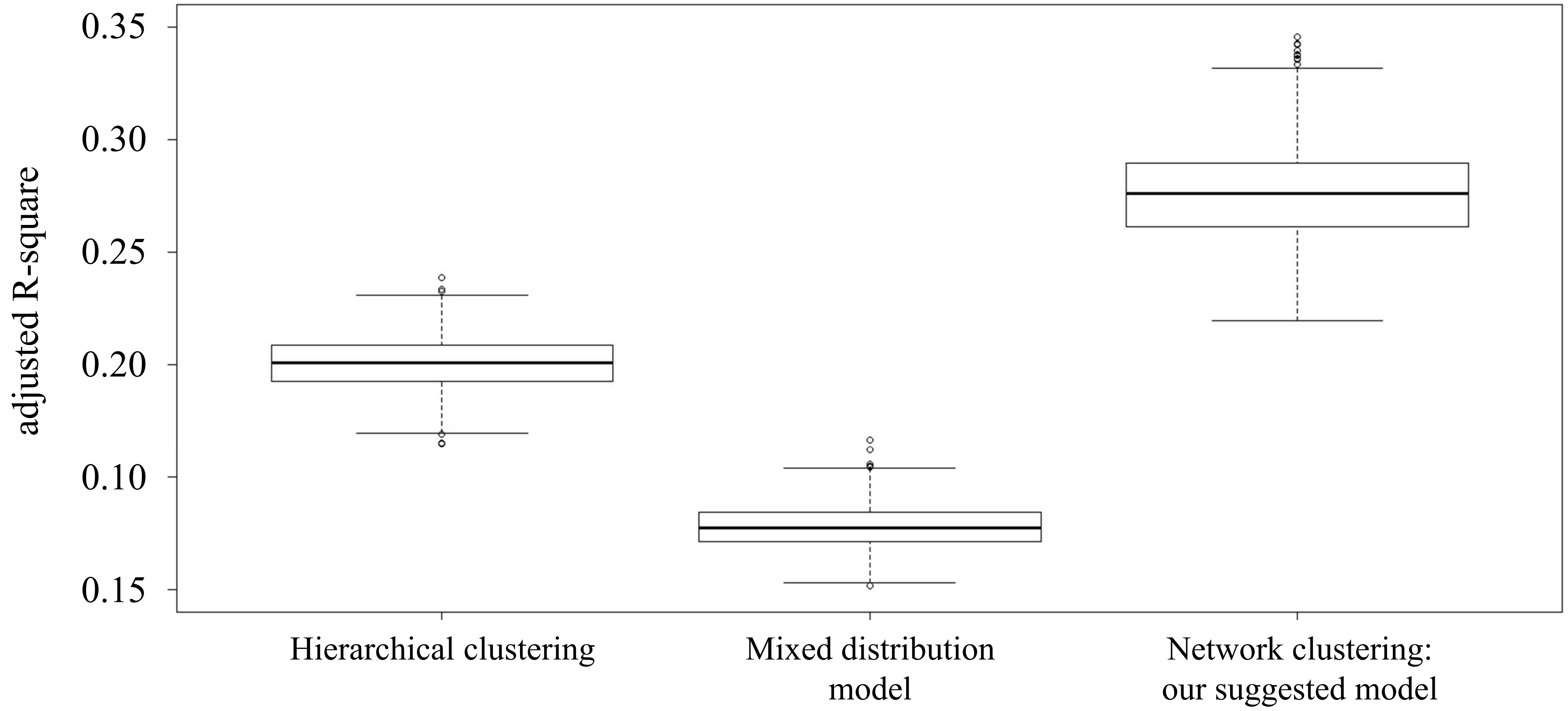

Supplemental Figure 2

Figure legends

Supplemental Figure 1. Confirmation of the number of initial components by sPLS.

According to existing methods, the optimal number of components via sPLS was estimated to be 2. We considered these two components as inexpedient for our purposes, as described in the manuscript (p. 15. Section 3.2.). Here, "component" was defined as a set of clusters of several correlated brain-region variables or biomarkers extracted by sPLS. Figure A and B show the number of variables (ordinate) in each initial component (abscissa) which were extracted as one component by sPLS. That is, the number of variables in one component corresponds to the size of the brain region (or set of biomarkers). The maximum number of variables included in each component of X did not change markedly with 10 or more initial components (Figure A), and the maximum number of Y variables did not change markedly with 5 or more initial components (Figure B). As such, we considered that even if we increased the number of initial components from 10 to many more, the size of each component extracted by sPLS would be stable. Figure C shows the number of components selected as relevant to AD by component selection using a logistic model. Similar to Figure A and B, the number of components selected as relevant to AD remained at 3, even if we increased the number of initial components (Figure C). Based on these results, we decided that the number of initial components for the logistic regression would be 10 in the present study.

Supplemental Figure 2. Result of numerical evaluation.

R square and MSE values calculated from bootstrap samples among the three clustering algorithms.
